# Visual number sense for real-world scenes shared by deep neural networks and humans

**DOI:** 10.1016/j.heliyon.2023.e18517

**Published:** 2023-07-24

**Authors:** Wu Wencheng, Yingxi Ge, Zhentao Zuo, Lin Chen, Xu Qin, Liu Zuxiang

**Affiliations:** a AHU-IAI AI Joint Laboratory, Anhui University, Hefei, 230601, China; b Institute of Artificial Intelligence, Hefei Comprehensive National Science Center, Hefei, 230088, China; c State Key Laboratory of Brain and Cognitive Science, Institute of Biophysics, Chinese Academy of Sciences, 15 Datun Road, Beijing, 100101, China; d CAS Center for Excellence in Brain Science and Intelligence Technology, China; e University of Chinese Academy of Sciences, 19A Yuquan Road, Beijing, 100049, China; f Key Laboratory of Intelligent Computing and Signal Processing of Ministry of Education, Hefei, 230601, China; g Anhui Provincial Key Laboratory of Multimodal Cognitive Computation, Anhui University, Hefei, 230601, China; h School of Computer Science and Technology, Anhui University, Hefei 230601, China

**Keywords:** Number sense, Deep neural network, Real-world scene, Group coding, Embedded representation

## Abstract

Recently, visual number sense has been identified from deep neural networks (DNNs). However, whether DNNs have the same capacity for real-world scenes, rather than the simple geometric figures that are often tested, is unclear. In this study, we explore the number perception of scenes using AlexNet and find that numerosity can be represented by the pattern of group activation of the category layer units. The global activation of these units increases with the number of objects in the scene, and the variations in their activation decrease accordingly. By decoding the numerosity from this pattern, we reveal that the embedding coefficient of a scene determines the likelihood of potential objects to contribute to numerical perception. This was demonstrated by the more optimized performance for pictures with relatively high embedding coefficients in both DNNs and humans. This study for the first time shows that a distinct feature in visual environments, revealed by DNNs, can modulate human perception, supported by a group-coding mechanism.

## 1 Introduction

Numerosity perception or number sense is the ability to estimate the number of potential objects/items in an environment without counting [[1], [2], [3], [4]]. It is one of the core functions of the brain that is shared by humans and several animal species [[5], [6], [7]], and provides a basis for advanced mathematical capacities in humans [8,9]. Moreover, it is assumed to be an innate and fundamental visual property that is not learned [[10], [11], [12]], as observed in newborn babies [13,14], chickens [15], and fish [16]. These species demonstrate the capacity to determine the number of objects they encounter, even without explicit feedback of performance or task requirements [17,18]. Recent advancement in artificial intelligence, particularly the application of deep neural networks (DNN), has allowed for the successful modeling of cognitive mechanisms [[19], [20], [21], [22], [23]]. For numerosity perception, deep neural networks can perform visual number distinction [24], and demonstrate number-selective units, similar to number-selective neurons [25] designed for object categorization [26]. Similar to human and animal new-born studies, number-selective units were identified in deep neural networks without training [27], indicating an innate mechanism associated with the structural properties of the network [28,29]. However, the number-selective responses in deep neural networks have been found to be sensitive to high-level organization rules [30], and learning is required to refine the initial numerosity sensitivity and thereby capture the statistical structure of the environment [31]. Notably, simple geometric figures, such as discs and rectangles, were extensively used as visual inputs in these studies. In animal studies, nourishment, such as natural prey [[32], [33], [34], [35], [36], [37], [38]], where spiders were used as a special example [39,40], cutting food into pieces [41,42], items (model eggs) that are closely related with survival [43], or objects of attachment identified in newly hatched chick experiments [44] was a relatively frequent choice of visual input, particularly in the beginning. When the animals were able to aim their behavior at more abstract visual inputs after some training, the stimulus was changed from food to featured objects, such as plastic discs [45] or visual figures such as landmarks, as observed in bee studies [46,47] and mosquitofish experiments [48], and finally to visual stimuli on computer screens. Using simple figures for visual stimuli aided in the exclusion of non-visual information, such as odor in food, which has been considered in studies of tortoises [41], and to control for potential magnitude factors that change with numerosity, such as luminance, perimeter, and area [49].

Well-defined visual stimuli combined with advanced neuroimaging techniques have allowed for the successful isolation of the influence of continuous magnitude from numerosity [50,51] and have confirmed the existence of an abstract number system that supports numerical skills [52]. However, the parametric design of visual inputs leaves one issue unsettled: the mechanism used by organisms to link the abstract sense of numbers to real objects in the environment. This is important because complex objects in real-world scenes are the contexts where they face survival [40]. For example, humans can perceive and distinguish between the complexity of real-world scenes where the number of objects is the dominant factor [53,54]. Furthermore, growing evidence shows that numerosity, representing an abstract quantity, is modulated by how we perceive visual inputs as perceptual objects or clusters [55], groupings [56,57], or other global constraints, such as connectedness [58,59]. Notably, objects in real-world scenes have potential statistical structures that organisms can learn during development [31].

Studies have shown that animals can perceive the number of prey and collaborators/competitors, and evaluate the relative size of these different groups of real objects to determine their predatory strategy [39,40], indicating the need for a high-level mechanism to link the abstract number and real objects with category information in the environment.

The perception of objects in real-world scenes is always entangled with the definition or category of an object. Category information is assumed to involve slow semantic processes [60]. However, new evidence shows that the human brain can rapidly extract the locations of semantically informative regions, as revealed by the representational similarity matrices (RSMs) of visual inputs and evoked brain responses [61]. In contrast to traditional analyses that compare the evoked potentials for two or more experimental conditions from individual electrodes, the RSMs of brain responses were determined by the pattern of evoked potentials of individual stimuli from all electrodes. The similarity of evoked patterns, in most cases measured as correlation coefficients between every pair of stimuli, represents abstracts about some of the innate characteristics of the stimuli [62], and can be compared with the RSMs calculated for semantic information or visual saliency from the same stimuli. The results revealed that although the link between physical saliency and brain responses emerged first, the link to semantic informativeness emerged immediately afterward, with a latency below 150 ms, as suggested by previous studies [60]. This demonstrates that the rapid processing of low-level global statistics and scene geometries can be detected through the pattern of brain activities representing sufficiently large cortical areas [61]. Using the same approach, the RSMs revealed the relatedness of object categories in a hierarchical manner from the responses of deep networks designed for categorization. The semantic concepts and nested structures were similar in the deep neural networks and WordNet, indicating an automatic abstraction of semantic relations by categorization [63]. These studies provide insight into the possibility that high-level constraints, such as numerical information in real-world scenes, could be detected by deep neural networks in a manner similar to semantic information, with group response patterns that go beyond single neuron sensitivity [25] or the population receptive field [64]. We speculate that the non-preferred responses in the group coding may behave as sidebands to carry information for numerical information.

Our hypothesis is when there are several potential objects in a real-world scene, these sidebands are different for each object, because their surroundings will never be the same. The sidebands could be accumulated and become detectable by normalizing the responses of the layer in relative to its maximum, resulting in an increased global intensity for scenes with more objects. In this study, we illustrate that the numerical information in real-world scenes can be detected by a pre-trained deep neural network, AlexNet, represented by the pattern of group activations that change with numerosity. The global activation intensity of the output units in the last fully connected layer increased with the number of objects in the scene. Meanwhile, activation variations decreased accordingly. We demonstrated that this change in patterns could be used to decode numerosity in real-world scenes, even when using a simple multilayer network, and showed remarkable robustness against noise. By testing several control conditions to check the contribution of object identities, their layout, and interaction with the surrounding environment, we determined how efficiently the objects in the scene could be represented by deep neural networks and scored as embedding coefficients, and determined how likely these potential objects were to be detected and to contribute to numerical perception in both deep neural networks and humans.

## 2 Results

**Fig. 1:**
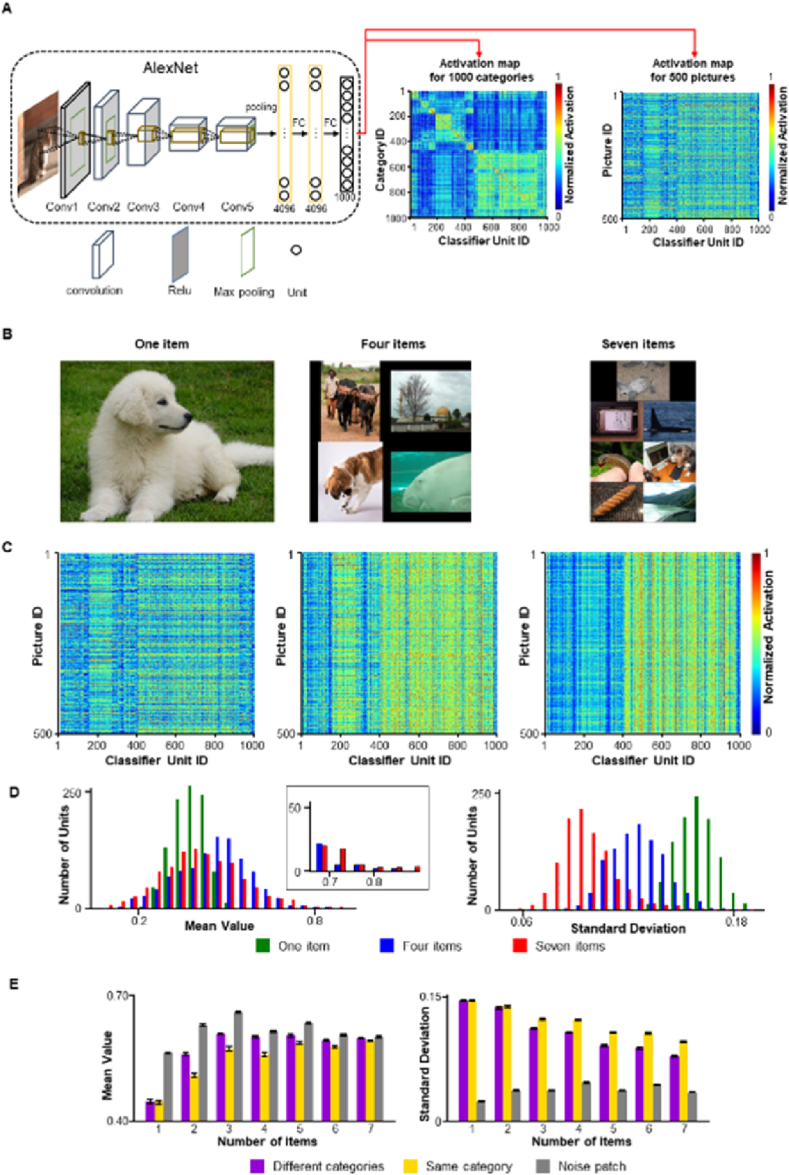
Real-world numerical information in pictures represented by the classifier units of the AlexNet. (**A**) Illustration of AlexNet and response pattern of its classifier units. In the activation map for 1000 categories, each line is the average response pattern of a category, showing a high response in the diagonal element that corresponds to the given category, with some off-diagonal co-activations. In the activation map for 500 pictures, each line represents the response pattern of one picture selected randomly from the dataset, showing co-activations as a general phenomenon shared by the pictures. (**B**) Sample mosaic pictures. Left: a single picture from ImageNet; middle: a mosaic picture composed of four items from different categories; right: a mosaic picture of seven items. (**C**) Activation maps for mosaic pictures with different numbers of items. From left to right: the general increased intensity from one to seven items and the shared co-activation pattern are presented as vertical lines across the pictures. (**D**) Left, with more items in the picture, more units pitch at higher activations; inset, the differences in the units with activation exceeding 0.7; right, significant shared co-activations increase with the number of items, characterized by more units having a smaller standard deviation of activation. (**E**) The 9th decile (90%) of the mean activation of the classifier units increased with the number of items, and the 1st decile (10%) of the standard deviations decreased accordingly. This was also observed for the pictures composed of items from the same category; however, pictures composed of noise patches showed a different profile. Error bars represent the standard deviation from the mean.

We first evaluated the pattern of activation in 1000 units of the output layer using a pre-trained AlexNet (Fig. 1A, left). This deep neural network was trained using the ImageNet dataset (ILSVRC2012), where pictures in the training and testing sets are organized into 1000 categories. When the responses of the 1000 classifier units for pictures in the testing set were averaged within categories, a category-specific activation matrix was obtained (Fig. 1A, middle). The maximum response for a given category is often observed in the diagonal cells of the activation matrix, indicating a correct or referred response [20]. There were less intense co-activations in the off-diagonal cells, which were supposed to be incorrect responses in other non-preferred units. When the responses of the 1000 classifier units were inspected picture-wise, as shown by randomly selecting 500 pictures from the testing set (Fig. 1A, right), the co-activations were more obvious. To test if these co-activations represent number information for real-world objects, presented as a kind of group pattern encoding, we generated mosaic pictures containing a different number of sub-blocks varying from 1 to 7 (Fig. 1B). A novel pattern of activation emerged gradually as the number of sub-blocks increased in the mosaic pictures (Fig. 1C, from left to right), and exhibited two main features. On one hand, the global activation of the output layer increased with the number of items, as shown in the histogram of mean activation of the 1000 classifier units (Fig. 1D, left, F (6, 6993) = 60.8, P < 0.001; 1 item < 4 items < 7 items, P < 0.001, corrected). There were more units with rather high activation (>0.7, Fig. 1D, inset) for pictures with 7 items than for those with a single item. On the other hand, certain units responded consistently to multiple pictures, forming a pattern of vertical lines (Fig. 1C, right). To evaluate the consistency of this shared activation, we measured the standard deviations across pictures. The results showed that the larger the number of items in the picture, the more classifier units showed smaller standard deviations across pictures (Fig. 1D, right, F (6, 6993) = 3119.7, P < 0.001; 1 item > 4 items > 7 items, P < 0.001, corrected). The same phenomenon was observed when the sub-blocks belonged to the same category (Fig. S1). However, when we replaced the sub-blocks with patches of white noise, although the mean activations increased with the number of items, the standard deviations also increased, which differed from the mosaic pictures (Fig. S2). We also tested this phenomenon using an untrained AlexNet, considering that number-selective units can spontaneously emerge in deep neural networks without training [27]. Our test results showed that the aforementioned phenomenon, featuring a relatively large mean activation and relatively small standard deviation for pictures containing more items, was not observable in the untrained AlexNet. To rule out the possibility that the effects exist only in rare cases, we repeated the procedure by randomly choosing 500 pictures from the mosaic picture pool 10 times. The 9th decile (90%) of the mean activations (Fig. 1E; left) and the 1st decile (10%) of the standard deviations (Fig. 1E; right) were measured for mosaic pictures composed of sub-blocks from different categories, sub-blocks from the same category, and noise patches, respectively. The results confirmed that pictures with more items in the activation of the 1000 classifier units had relatively high intensity and relatively small variations.

### 2.1 Numerosity in real-world pictures represented by activation patterns of deep neural networks

**Fig. 2:**
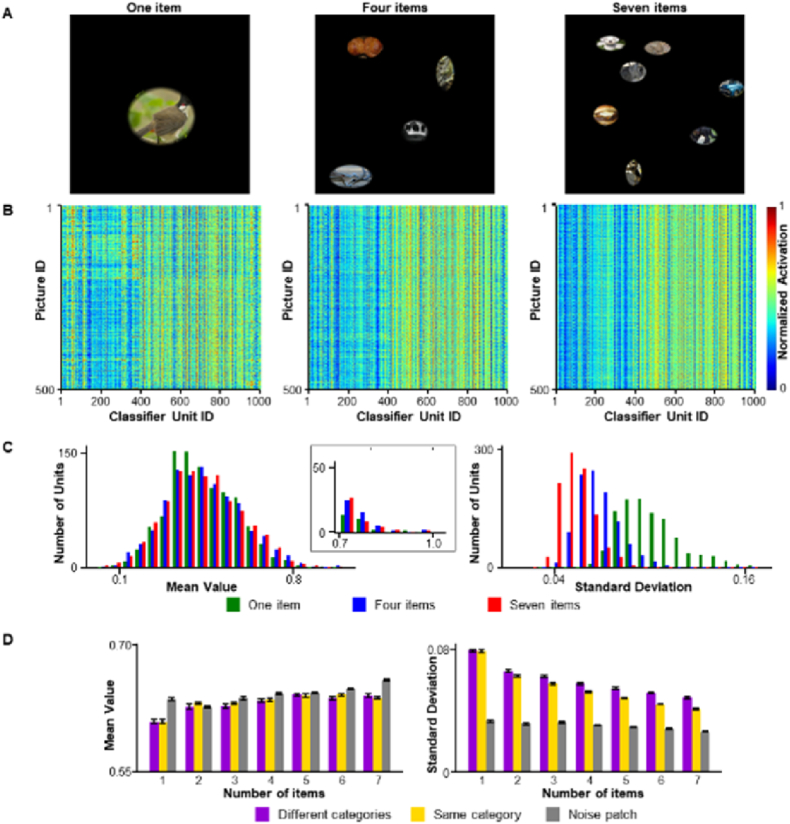
Numerosity represented by group activations for real-world items. (**A**) Sample visual stimuli with real-world items. Real-world items are obtained from ImageNet and clipped to elliptical areas with soft edges. The size of the items in the composed picture was controlled by considering the number of items and total area of all items. The density of items was balanced to a certain degree in the different datasets. Left: one item; middle: four items from different categories; right; seven items from different categories. (**B**) Activation maps for pictures with different numbers of items. From left to right, the intensity increased from one to seven items and the shared coactivation pattern as vertical lines across the pictures was obvious. (**C**) Left, with more items in the picture, more units pitch at higher activations; inset, the differences in the units with activation exceeding 0.7; right, significant shared co-activations increase with the number of items, characterized by more units having a smaller standard deviation of activation. (**D**) The 9th decile (90%) of the mean activation of the classifier units increased with the number of items, and the 1st decile (10%) of the standard deviations decreased accordingly. This is also true for the pictures composed of items from the same category and those composed of noise patches. Error bars represent the standard deviation from the mean.

As the mosaic pictures were composed of a rigid layout, the effects found here may originate from uncontrolled factors, such as the density or size of the sub-blocks. In previous studies involving number-selective units in deep neural networks often involved the use of simple geometric figures such as dots or squares [[24], [25], [26], [27]], with careful design of the layout of the display and the size of the items to reduce the effects of these factors. Using the same approach, we designed images comprising real-world items from the ImageNet dataset. Objects in the ImageNet dataset were clipped using the inscribed ellipse of their bounding box [31] and rescaled to the appropriate size, followed by the application of a Gaussian filter along the edge for smooth fading out to the black background. The size of the ellipses was carefully determined to keep the total area approximately matched from one to seven items with reasonable random variants between objects in a single picture (Fig. 2A).

Each composed picture was divided into 4 × 4 virtual grids, and objects were randomly assigned to the grids. The precise position of the object was jittered within the grid to compensate for the tradeoff between the density and number of objects. Similar to the mosaic pictures, the activations of the 1000 classifier units were evaluated by randomly choosing 500 pictures from the datasets containing 1–7 items, and the procedure was repeated 10 times. An almost identical pattern was observed in these pictures (Fig. 2B); the mean activation was larger for pictures with more items, whereas the standard deviation was significantly smaller (Fig. 2C). We also composed two control versions of the pictures; the items are from the same category (Fig. S3) and the ellipse was filled with white noise (Fig. S4) and yielded almost the same results. The 9th decile (90%) of the mean activations (Fig. 2D left) or 1st decile (10%) of the standard deviations (Fig. 2D right) revealed that regardless of whether the items are from the same or different categories, the mean activations increase with the number of items (for different categories, F(6, 63) = 3187.1, P < 0.001; for the same category, F(6, 63) = 1588.7, P < 0.001) and the standard deviations decrease accordingly (for different categories, F(6, 63) = 4418.3, P < 0.001; for the same category, F(6, 63) = 2848.5, P < 0.001). For the ellipsoidal noise patches, the tendency is the same (mean activation, F(6, 63) = 1560.9, P < 0.001; standard deviation, F(6, 63) = 1275.3, P < 0.001), but the absolute values differ from the other two conditions.

### 2.2 Number of objects in real-world scenes represented by activation patterns

**Fig. 3:**
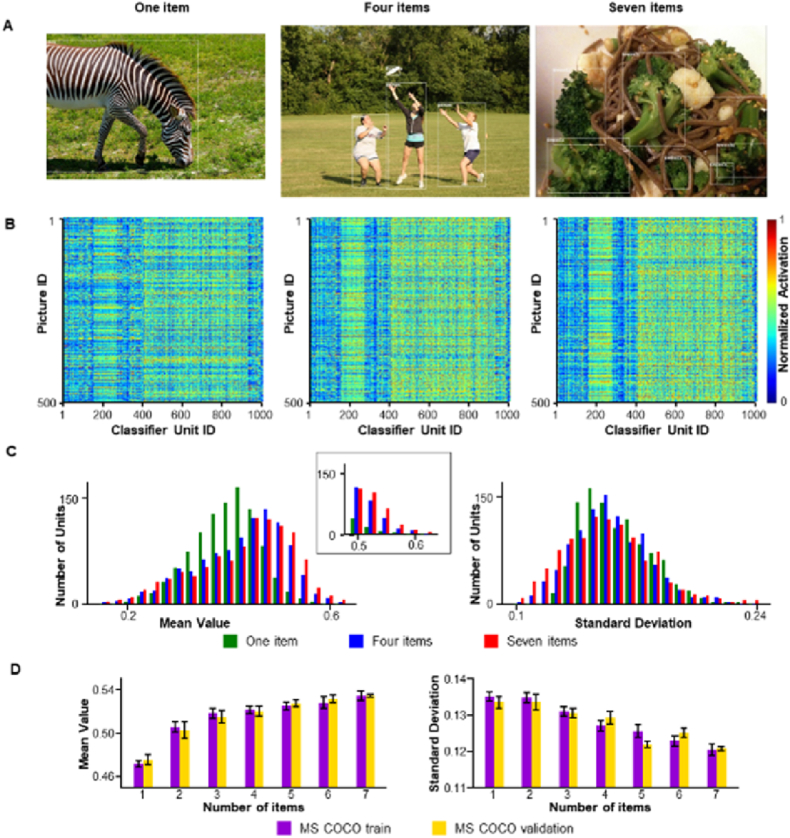
Group activations increase with the number of potential objects in natural scenes. (**A**) Sample pictures from MS COCO dataset. Left: one potential object; middle: four potential objects; right: seven potential objects. The bounding box for each object is illustrated as a white rectangle overlaid on the picture. (**B**) Activation maps for different numbers of objects. From left to right, the pattern shared across different pictures and the intensity increases with the number of potential objects. (**C**) For these natural scenes, the distribution of activations yielded relatively high mean values and relatively small standard deviations for pictures with more potential objects, similar to the mosaic pictures. (**D**) The 9th decile (90%) of the mean activation of the classifier units increased with the number of items, and the 1st decile (10%) of the standard deviations decreased accordingly. Error bars represent the standard deviation from the mean.

It is unclear whether our new findings depend on the segregation of certain regions from the background, either in a rigid mosaic form (Fig. 1B) or in more naturally ellipsoidal patches (Fig. 2A), through their interactions with the contents within these regions. To answer this question, we evaluated the activation pattern of a pre-trained AlexNet for real scenes in the MS COCO dataset. The MS COCO datasets contain approximately 328 K images, where bounding boxes label common objects in context with a category tag. From the MS COCO train dataset, pictures with one bounding box (bbox) were selected as Group 1, pictures with 2 bounding boxes as Group 2, in that order until pictures with 7 bounding boxes as Group 7 (Fig. 3A). We randomly selected 4000 pictures from each group to generate a training set (COCO_train). The activations in the 1000 output units of AlexNet were evaluated for all pictures (4000 × 7 pictures). The results show the same phenomenon as that observed in the mosaic pictures. The normalized activations of the output units increased with the number of bounding boxes (objects) in the pictures, and the off-diagonal activations shared across pictures as vertical lines were more visible for pictures with more objects (Fig. 3B, left to right). In addition, the distribution of the mean activation showed that the activation of the output layer was larger for pictures with 7 items (Fig. 3C, left, F(6, 6993) = 18.9, P < 0.001; one item < 4 item < 7 items, P < 0.001, corrected). In addition, more units had rather high activation (>0.7; Fig. 3C, inset) for pictures with 7 items. For consistency among the co-activations across pictures, the standard deviations of the activation were relatively small for pictures with more objects (Fig. 3C, right, F(6, 6993) = 13.3, P < 0.001; 1 item > 4 items > 7 items, P < 0.001, corrected). When evaluated with the 9th decile (90%) of the mean activations (Fig. 3D, left) or the 1st decile (10%) of the standard deviations (Fig. 3D, right), unipolar changes consistent with the number of items were identified.

Following the same procedure, we prepared an additional 7 groups of pictures for validation (COCO_val), each with 150 pictures from the MS-COCO test dataset. The activations for this dataset were also evaluated (Fig. S5). The results confirmed the same pattern as that found in the COCO_train dataset: mean (F(6, 6993) = 6.9, P < 0.001; 1 item < 4 items, P < 0.01; 4 items < 7 items, P < 0.001, corrected) and standard deviation (F(6, 6993) = 818.8, P < 0.001; 1 item > 4 items > 7 items, P < 0.001, corrected).

### 2.3 Decoding numerosity in real-world scenes

**Fig. 4:**
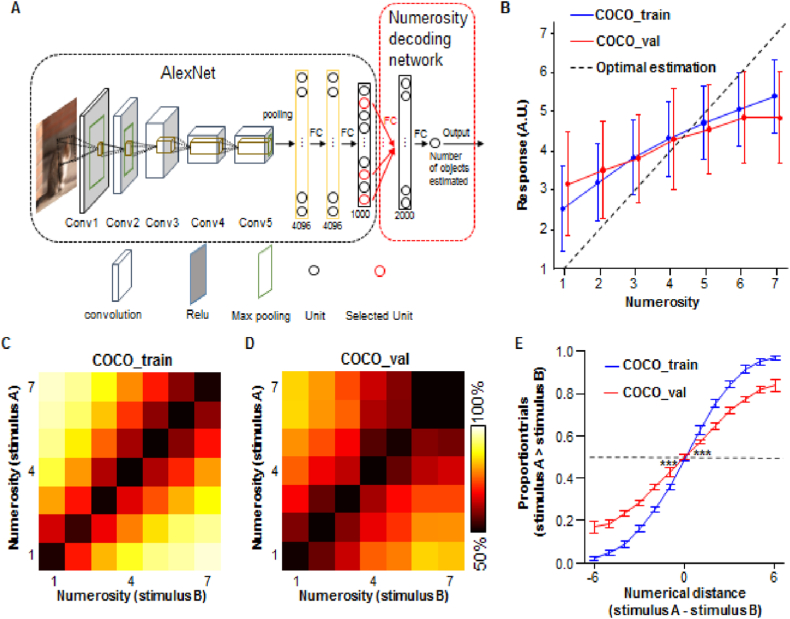
Decoding numerosity in real-world scenes and human-like performance in comparison tasks. (**A**) Architecture of the AlexNet and the activations of the selected units (red) in its output layer served as inputs for the simple decoding network that followed. (**B**) Responses of decoding network. Blue: results for the training set (COCO_train); red: results for the testing set (COCO_val); black: assumed optimal responses. The results were less than optimal for numbers 6 and 7. (**C**) Performance matrix of numerosity comparison across different combinations of numbers 1–7 using pictures from the training and (**D**) testing set. (**E**) The psychometric function represents a typical S-curve with a smaller slope for a larger numerical distance between the two pictures, similar to humans. The dotted line indicates a 50% chance level. Error bars represent the standard deviation from the mean. ***, P < 0.001. (For interpretation of the references to colour in this figure legend, the reader is referred to the Web version of this article.)

It is unclear whether numerosity in real-world scenes, represented by the activation pattern of the output units, can support a type of visual number perception in a DNN. To test this possibility, we designed a simple network to decode the numerical information in pictures. Active units were selected from the 1000 output units with the following criteria: presence of increasing or decreasing activations along with the number of objects in the pictures; the difference in activations with a p value below 0.1 for every consecutive number of pairs. Based on the activation of the pictures in the COCO_train dataset, there were 48 increasing units (1 item < 2 items < … < 7 items) and 13 decreasing units (1 item >2 items > … > 7 items). Considering the activations in these 61 units as inputs, the decoding network had a fully connected hidden layer of 2000 units and a fully connected final unit (Fig. 4A). The response of the final unit is the decoding result, which was assumed to be the estimated number of objects in the picture. We trained the decoding network using the COCO_train dataset (4000 × 7 pictures) and evaluated it using both the COCO_train and COCO_val datasets (150 × 7 pictures).

The results showed a significant positive relationship with the number of objects in the picture (Fig. 4B) for both the COCO_train (F (6, 27,993) = 4571.3, P < 0.001) and COCO_val (F (6, 1043) = 45.5, P < 0.001) datasets. Moreover, the response levels of the decoding network are larger than the optimal estimates for relatively small numbers (1 and 2). In contrast, for larger numbers (six and seven), the responses are significantly smaller than assumed (P < 0.001, corrected), similar to human behavior [58]. Although the datasets had no main effect (F(1, 29,036) = 0.36, P = 0.55), there was significant interaction between numerosity and the dataset (F(6, 29,036) = 21.6, P < 0.001). Post hoc analysis indicated that the responses to each pair of nearby numbers are significantly different for the COCO_train dataset (P < 0.001, corrected). The same tests failed for the COCO_val dataset, except for number 3 vs. 4 (P < 0.05, corrected). The COCO_val dataset passed most of the tests at a numerical distance of 2 (1 vs. 3, 2 vs. 4, 3 vs.5, P < 0.001; 4 vs. 6, P < 0.01; 5 vs. 7, P = 0.53).

We then adopted a numerosity comparison task from human studies [31], to evaluate the decoding network performance. One hundred pictures were randomly selected from one of the seven groups as stimulus A, and their decoding responses were compared in pairs with those of another hundred pictures (stimulus B) randomly selected from the same or other groups. The same procedure was applied across all combinations of numbers 1 to 7, resulting in a 7 × 7 matrix, and repeated 10 times. The results showed that the further away the decoding network was from the diagonal elements (larger numerical distance between stimuli A and B), the more optimized its performance was for both the COCO_train and COCO_val datasets (Fig. 4C and D). When the performance was measured as a psychometric function of the numerical distance, a typical S-curve, similar to that found in human perception, was obtained (Fig. 4E). Notably, although the performance for the COCO_train dataset was more optimized at every numerical distance (P < 0.002, corrected; except for numerical distance equal to zero), the performance for the COCO_val dataset significantly exceeded the 50% chance level, even when the numerical distance was 1 or -1 (P < 0.001, corrected). This indicates that visual number sense can be generalized from the training dataset to other real-world scenes and may provide a reference for tasks such as numerosity comparison.

**Fig. 5:**
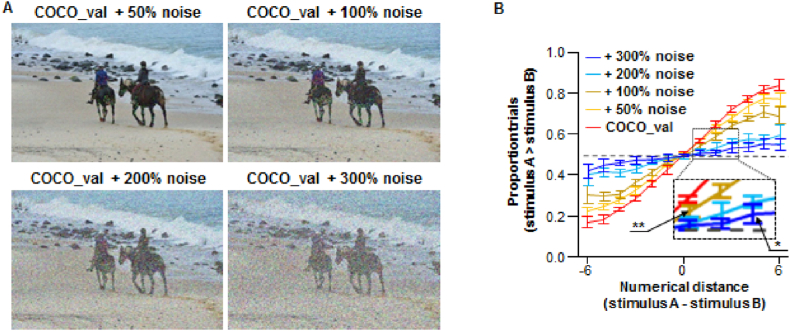
Robustness of decoding against white noise. (**A**) Sample pictures with different white noise weights. (**B**) The decoding network performed significantly better than chance level in the comparison task when the numerical distance is only 1 for pictures with 100% and 300% noise at a numerical distance of 3 (inset); however, the performance decreased with additional noise. The dotted line indicates a 50% chance level. Error bars represent the standard deviation from the mean. *, P < 0.05; **, P < 0.01.

Subsequently, we tested the noise robustness of the decoding network by overlaying white noise on the COCO_val dataset using different weights (Fig. 5A). As an abstract property emerging from visual inputs [50], visual number sense is assumed to be insensitive to external or internal noise [65]. Although adding noise to the inputs gradually affected the performance negatively, the shape of the psychometric function was adequately kept until 200% noise was applied (Fig. 5B). The resistance against noise is so strong that the performance was significantly above chance level when the numerical distance was 1 or -1 (P < 0.01, corrected for 100% noise). Even in the worst condition tested (300% noise), the decoding network successfully distinguished the two inputs when the numerical distance is ≥3 (P < 0.05, corrected).

### 2.4 Contributions of foreground/background information and separation

**Fig. 6:**
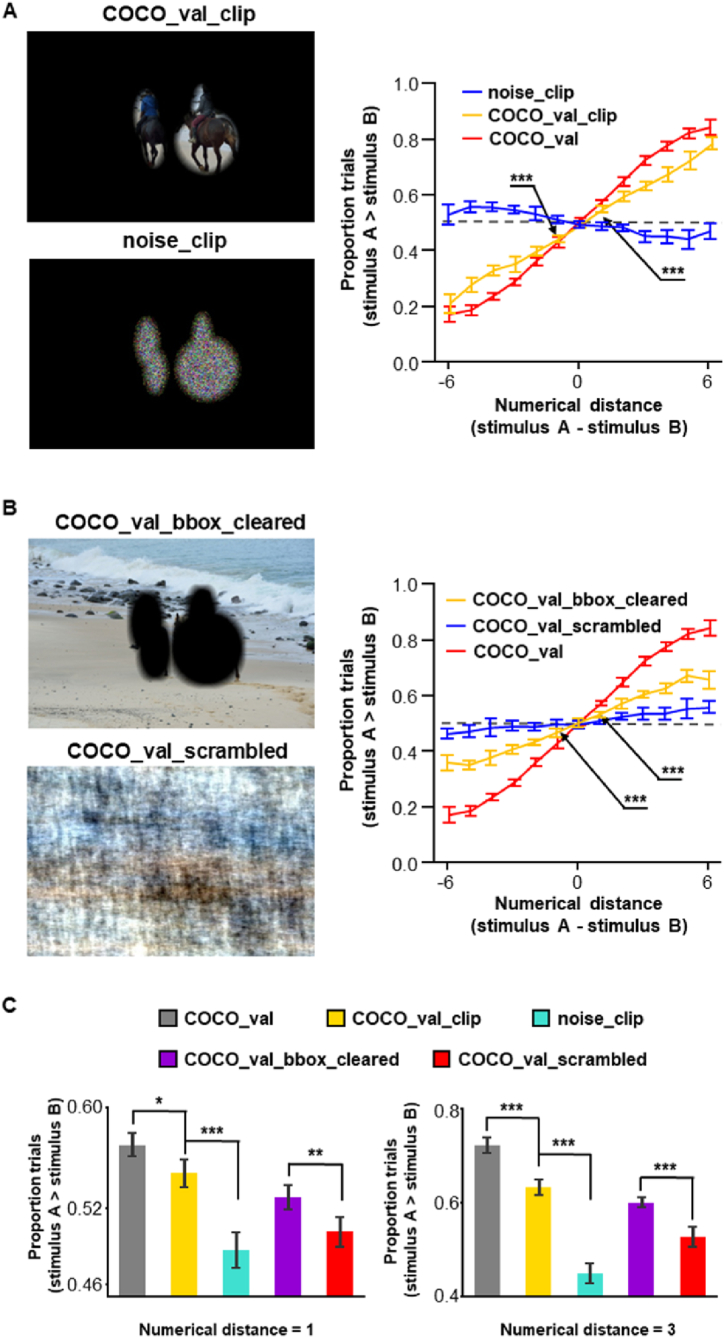
Foreground and background factors contribute to the visual number sense. (**A**) By clearing the contents outside the bounding boxes, a degraded performance demonstrated the contribution of details in the background. The abstract numerosity based on foreground-background separation cannot explain the behavior of our decoding network, shown as almost none, even reversed effect, when bounding boxes were filled with white noise. (**B**) The essential role of foreground objects was demonstrated after the bounding boxes were cleared, particularly compared to the phase scrambled version of the original pictures. (**C**) Performance in the COCO_val_bbox_cleared condition was more optimized than that in the scrambled condition. The performance of the original dataset and the COCO_val_clip condition demonstrated the contribution of the objects in the foreground, while the difference between the two indicated potential supplementary contribution of the background details. The dotted line indicates a 50% chance level. Error bars represent standard deviation from the mean. *, P < 0.05; **, P < 0.01; ***, P < 0.001.

The use of real-world scenes as inputs provides ecological details; in addition, real-world scenes are relatively compatible with real-world tasks. However, the potential mechanisms involved in the process are not easily identified compared with those for the use of simple geometric figures as inputs. One of the most notable distinctions is the rich information in the background of real-world scenarios. We tested whether the background information played a role in visual number sense. The background was set to black for the pictures in the COCO_val dataset, whereas the contents in the elliptical region were preserved (COCO_val_clip; Fig. 6A, left). The performance of the decoding network remained remarkable for these pictures, despite not being as good as that of the original pictures (Fig. 6A, right). For example, it was 66.38% vs. 72.28% correct at a numerical distance of 3 (Fig. 6C, right, P < 0.001, corrected), and both were more optimized than the chance level (P < 0.001, corrected).

Given the significant decrease in performance, the contribution of the background is impressive. However, segregation of the elliptical region from the background may play a role in this performance. To test this hypothesis, we filled the elliptical regions with white noise in the modified pictures (noise clip, Fig. 6A, left). The results showed that the perception of numerosity disappeared (Fig. 6A, right). Therefore, background information was involved in the visual number sense observed in the deep networks, and the foreground/background separation could not explain the performance of the comparison task.

Subsequently, we evaluated the contribution of the objects in the foreground by shading the elliptical regions black and leaving the background intact (COCO_val_bbox_cleared; Fig. 6B). The performance of the decoding network was significantly attenuated (Fig. 6B, right, P < 0.01, corrected) compared with that of the original pictures. Nevertheless, the performance exceeded that of the chance level (Fig. 6C).

To obtain a fair baseline condition without introducing extra foreground/background separation, we generated a phase-scrambled version of the original dataset (COCO_val_scrambled; Fig. 6B, left). Phase-scrambled pictures are supposed to have low-level details with an amplitude spectrum that is intact, whereas the high-level visual information of natural images is disrupted [66]. The decoding performance decreased to chance level (at least for a numerical distance of ≤3) for the phase scrambled pictures. Notably, the pictures in the COCO_val_bbox_cleared condition exhibited significantly more optimized performance than chance level (Fig. 6C, P < 0.001, corrected).

Our results show that the main source of decoding power originates from the high-level visual information carried by the foreground objects, and background information plays a complementary role (Fig. 6C).

### 2.5 Embedding representation underpins the variation in perceptual performance

The abovementioned high-level abstract numerosity is represented in the pattern of group activation of the output units. In contrast to the recently reported tuning properties of number-selective units revealed by simple geometrical figures [[24], [25], [26], [27]], this pattern of group activation in the output layer can be conceptualized as manifolds in high-dimensional space as a type of embedding representation. Generally, well-trained neural networks designed for object classification that assign pictures to given categories will finally converge to the same high-dimensional representations [67]. As the original pictures were decoded better than the noise clip pictures without object identity, a straightforward prediction is made: the better the visual inputs assigned to the pre-trained categories (embedded into the manifolds), the better the performance of visual numerical perception.

**Fig. 7:**
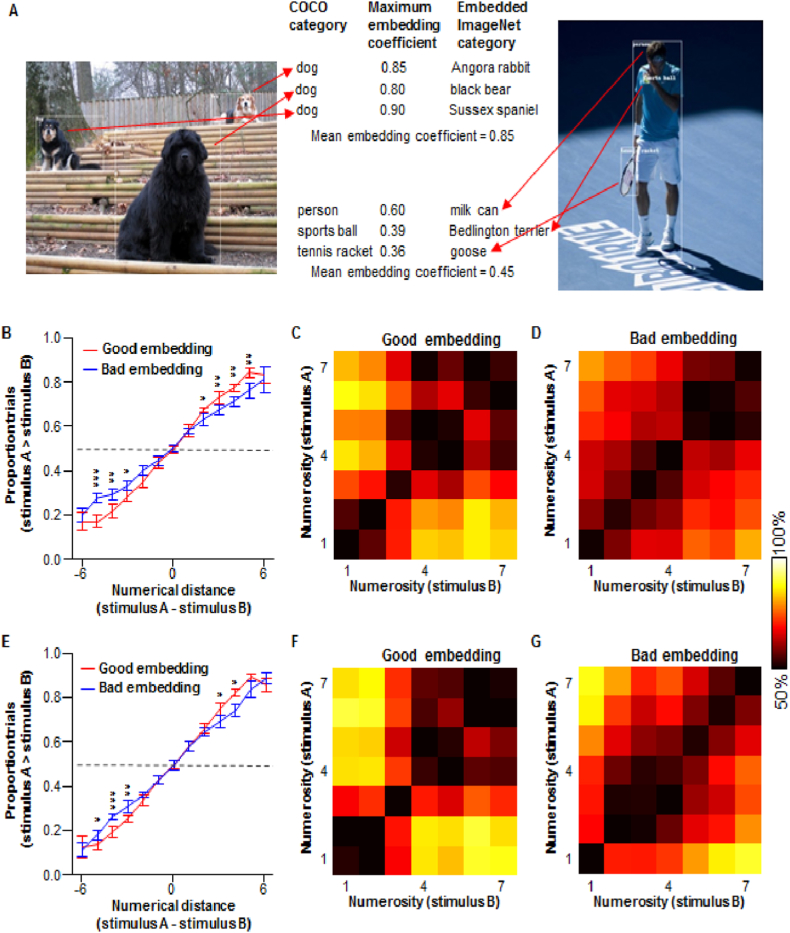
Superiority of embedding representation affects the visual number sense. (**A**) Superiority of embedding representation was determined by the maximum embedding coefficient of every bounding box in a picture. Left: an example of remarkable embedding with a high mean coefficient. Right: an example of inferior embedding. Notice the embedded ImageNet categories differ from what the objects are supposed to be. (**B**) The superior embedded subset performed better than the inferior embedded subset, as revealed by the psychometric functions. The difference could also be observed easily in the performance matrix of numerosity comparison across different combinations of numbers in (**C**) and (**D**), for superior and inferior embedding, respectively. (**E**) The category-based embedding coefficient also demonstrated the difference between superior and inferior embedding. The distinctions in the performance matrix between (**F**) and (**G**) were similar to those shown between (**C**) and (**D**).

To test this prediction, we measured how well the pictures in the COCO_val dataset were embedded in the manifolds of the pre-trained AlexNet. The content in each bounding box was cropped for each picture and served as the input to AlexNet. The activations in the output units for each bounding box were compared with those of the 1000 categories to determine the optimal match (Fig. 1A, middle). Correlation coefficients, as absolute measures of the quality of embedding [68], were calculated between this bounding box and all categories. The category with the maximum correlation coefficient was selected as the embedded category, and the coefficient was assigned to the bounding box. For a given picture, the embedding coefficient was determined by averaging the coefficients across all inherent bounding boxes (Fig. 7A). All the pictures in the COCO_val dataset were sorted in the order of their embedding coefficients. We selected the top 50 (referred to as the superior embedded subset) out of the 150 pictures in each of the 7 groups (Fig. 7A; left), and the decoding performance was evaluated for this subset, as well as for the entire dataset. We also applied the same procedure to the bottom 50 pictures (referred to as the inferior embedded subset) (Fig. 7A; right). The results show that the performance was fairly remarkable for both subsets, similar to that of the entire dataset (Fig. 7B). More importantly, the superior embedded subset performed significantly better than the inferior embedded subset (Fig. 7B–7D, P < 0.05, at numerical distances of 2, P < 0.01 at numerical distances of 3, 4, and 5). This demonstrates that based on the numerical information they possess, pictures can be more easily discriminated against each other if the embedded representation of the objects within these pictures fits better with the internal manifolds of the pre-trained deep neural network.

Notably, the best-fit embedded (ImageNet) category for a given bounding box is sometimes not the proper match of its category label given by the MS COCO dataset (Fig. 7A, middle, especially for the inferior embedded subset). Although some of the mismatches could be interpreted in the context of category vs. super category [63] or explained by similar features that are shared across several categories, whether the contribution of embedded representation to visual number sense is merely based on detailed visual features of the objects, rather than their abstract identity/category, is a concern. To address this concern, we defined a category-based embedding coefficient. The bounding boxes (4200 in total for the 150 × 7 pictures) were sorted into 80 categories according to their MS COCO labels. For each bounding box in a given picture, the embedding coefficient was replaced with the mean coefficient of the category to which it belongs. This manipulation allowed for the determination of the coefficient of each picture via the abstract identity of the bounding boxes by averaging across the bounding boxes. The pictures were sorted into superior and inferior embedded subsets according to the category-based coefficients. Pictures with superior embedding exhibit more optimized performance than those with inferior embedding (Fig. 7E–G) at a numerical distance of ≥3 (P < 0.05, corrected). This analysis demonstrates that the superiority of the embedded representation of a real-world scene depends on the visual appearance of objects, as well as their abstract identities/categories.

### 2.6 Humans shared the embedding constraints on visual number perception with deep neural networks

The modulation of the superiority of embedding in visual number sense, revealed by group activations in the DNN, presents a clue for linking the embedded representation of object identities with numerosity in real-world scenes. However, it is unclear whether these mechanisms substitute human visual perception in real-world experiences. As the semantic relations that emerged in the DNN share an almost identical hierarchical relatedness with WordNet, a database based on human language [63], we speculate that (a) human participants have a visual number sense for real-world scenes and (b) the superiority of embedding plays a role in human perception.

**Fig. 8:**
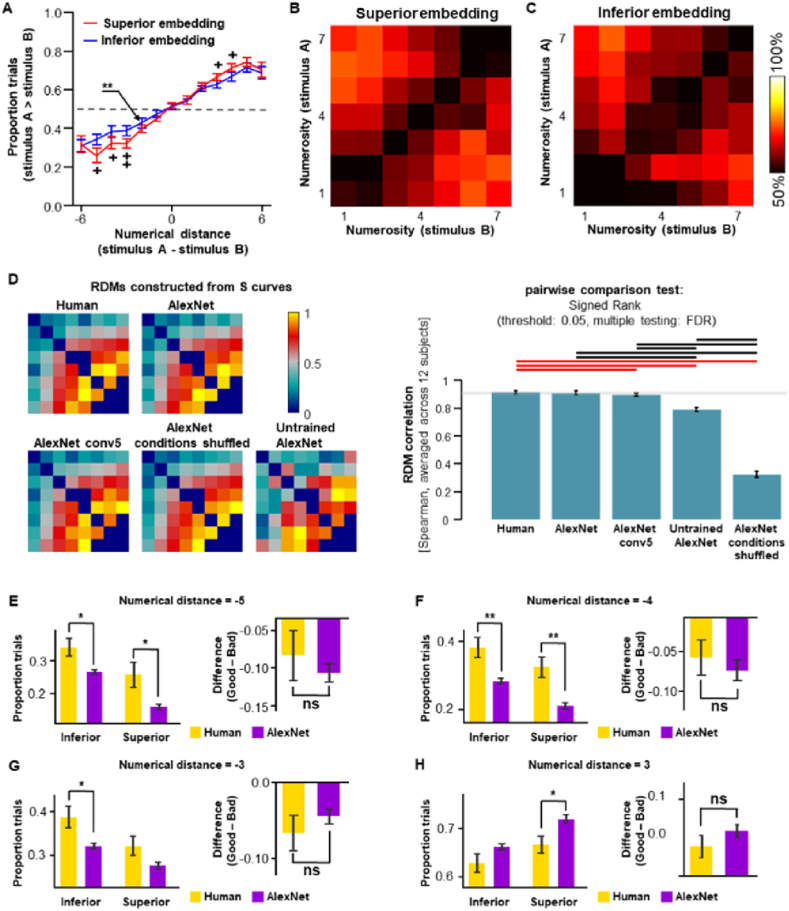
Humans and deep neural networks share the same embedding constraints on visual number perception. (**A**) Similar to AlexNet, human participants (N = 12) showed more optimized visual number perception for the superior embedded real-world scenes than the inferior embedded subset. The difference was also shown in the performance matrix in (**B**) and (**C**), for superior and inferior embedding, respectively. (**D**) Representational similarity analysis for human behavioral performance and AlexNet. Left, representational dissimilarity matrix constructed from S curves of superior and inferior embedding conditions. Right, human performance is almost identical to that of AlexNet, and significantly differs from the results of the best layer (conv5) in terms of number selectivity, AlexNet with conditions shuffled, or the untrained AlexNet with random weights (pairwise comparison shown in red). (**E**) Despite AlexNet showing more optimized performance than humans, the advantage of superior embedding over inferior embedding was essentially the same when the numerical distance was −5, (**F**) −4, (**G**) −3, (**H**) 3. Error bars represent standard errors. +, P < 0.05, uncorrected; ++, P < 0.02, uncorrected; *, P < 0.05, **, P < 0.01. N = 12. (For interpretation of the references to colour in this figure legend, the reader is referred to the Web version of this article.)

To test these hypotheses, we applied the same superior and inferior embedded subsets in a numerosity comparison task for humans using a procedure similar to that used in our previous studies [58]. Two pictures were briefly presented on the screen's left and right hemifields, and the participants were instructed to identify the picture with more “items or something.” The participants were reminded to base their decision on their feelings and definition of the object, and maintain stability in criteria throughout the test. Reliable performance matrices and psychometric functions were obtained from 12 of the 17 participants of the study; Fig. S6 shows the individual data, and Fig. S7 the data from the 5 participants excluded from the study. Consistent with previous studies on the scene complexity, our results showed that humans could discriminate numerosity in real-world scenes at a numerical distance of 2 at least (Fig. 8A, indicated by asterisks), and the psychometric functions are similar to those of AlexNet.

Remarkably, the superior embedded subset had significantly more optimized performance than the inferior embedded subset (Fig. 8A–C, P < 0.05 at a numerical distance of 3, 4, −4, and −5, P < 0.02 at a numerical distance of −3, uncorrected) in human participants, similar to what was observed in the AlexNet. We compared the results from human participants with those yielded by AlexNet through representational similarity analysis [62] at the condition and picture-wise levels using the RSA toolbox [69]. By filling the S-curve of the comparison task into a representational similarity matrix (RSM), the innate characteristics of human performance were compared with those of AlexNet and three other control conditions (the last convolutional layer conv5 of AlexNet, condition-shuffled AlexNet and untrained AlexNet; Fig. 8D, left). The results showed that the performance of the human participants is similar to that of AlexNet; however, the two differed significantly from the three control conditions (Fig. 8D; right). The same results were confirmed at the picture-wise level for items 1–7 (Fig. S8). When the results from human participants and AlexNet were compared directly, the number of advantages of superior embedding over inferior embedding is almost the same in the two types of cognitive agents (Fig. 8–H, right panel), despite AlexNet generally outperforming human participants in numerical distinction (Fig. 8–H, left panel).

## 3 Discussion

Using a pre-trained DNN designed for category classification, we showed that numerosity in real-world scenes can be represented by the activation pattern of units in the classifier layer. We found that the identity information carried by the objects in the scenes played a major role, and the background contributed to numerosity perception. When the contents of the visual inputs fit the internal representations in the DNN, indexed as relatively high embedding coefficients, more optimized performance was achieved in a numerosity comparison task. We applied the same task and sets of stimuli to the human participants. The mechanisms of group coding and embedded representation in a DNN can be generalized to probe human visual perception.

Although only a few studies have focused on the human perception of numerosity in real-world scenes, increasing evidence shows that certain global statistics in real-world scenes or their layout properties, such as asymmetry or alignment, can be detected by the human visual system, with a latency similar to that of low-level features [[70], [71], [72]]. This latency is shorter than that previously assumed for high-level processing, which follows the perception of low-level features, indicating a quick grasp of global characteristics in real-world scenes. To a certain degree, the potential objects or types of relevant statistical identities that come with each unit are required to emerge from the background before the global information or layout of the scenes can be perceived. This capacity implicitly leads to numerosity perception in the scenes, because subitizing is one of the central abilities for number perception [73]. Perception of the complexity of real-world scenes has been demonstrated in humans. The first major dimension supporting the complexity perception of scenes is the number of objects, followed by their layout [54]. Evidence suggests that past experiences with an object also play a role in the perception of visual complexity [55]. These results imply that although the extent to which a part of a scene should be perceived as an object is arguable and may change with development or learning, humans can perceive numerical information in real-world scenes. As this global information is processed rather quickly, parallel to low-level features, counting mechanisms are unlikely to have been applied.

Recent advances in artificial neural networks have led to the discovery of several sense units in deep neural networks designed for object recognition or classification. In addition, number sense can emerge from deep neural networks designed to detect numerosity with or without learning. However, despite the differences in these two approaches at face value, that is, one emphasizes the selectivity of single units and the other relies on the structural connection and weighting between units, both approaches represent numerosity by the activity of single units, either the selective or final reporting ones. The present study found that numerosity could be represented as a pattern of group activity in the category layer. The global intensity of the category layer units increased with the number of objects in the scene, whereas the variations across different scenes decreased. Certainly, some of the units had stronger effects than others, we used them in the simple decoding network. A theoretical explanation of the group coding is that it is impossible for a DNN, such as AlexNet, to convey to its optimized state for a real-world dataset; that is, when considering the activations of the category layer for a given group of input pictures, only the unit corresponding to this category was activated as 1, leaving other units as 0. However, in real applications of AlexNet, every unit shows some activation of the input picture, and the categories corresponding to the top unit or the top five units are selected as outputs. These coactivations in non-preferred units allow us to decode relative information, such as semantic relations and numerosity, in real-world pictures by group coding, in addition to category classification according to the design of the DNN. This group coding can be summarized as embedded representations, which, for pictures in a given category, should stay around their group center with minimal variations and maintain a distance from other centers [67]. This is consistent with the high-dimensional clustering framework suggested by Topological Data Analysis [74] or topologically reserved structures in the feature space [75] with reasonable tolerance spacing [76].

The non-optimal response of AlexNet to real-world scenes suggests a possible confounding factor for the phenomenon we observed here. Instead of representing the potential number of objects, the pattern of activation represents the likelihood of the existence of a certain category in the picture. However, the decoding performance of pictures composed of objects from the same (Fig. S3) or different categories (Fig. 2A) did not show significant differences (Fig. S9). Regardless of whether the phenomenon concerns the certainty or uncertainty of the category, these two conditions should differ, which contradicts the current results.

The experience dependency revealed by the contrast between the pre-trained AlexNet and untrained network suggests that the group coding of numerosity for real-world scenes is something between the number of selective neurons and a particular deep network designed for number discrimination. With repeated exposure to pictures with rich structural information, the group coding of numerosity automatically emerged in the deep network, although the training is not directly number-oriented. This emergence may be rooted in the process of segmenting objects from the background, linking it to an implicit assumption for numerical perception: the inputs could be segregated by some kind of criterion into isolatable units [73]. This is obvious in conditions with simple visual figures, such as dots or squares, but is more important when an abstract representation of numbers has been considered. For example, Barth et al. (2003) demonstrated that either visual or auditory input streams or cross-modality inputs could represent numerosity, thereby excluding the influence of local features at the perceptual level and pinpointing the existence of a pure abstract number system [77]. However, to make the cross-modality system work, there are prerequisites. The two sensory channels automatically segregate the input stream into countable units: a small dot in the visual domain and a pure tone beep in the auditory domain. This is essentially related to the definition of an object, which, as pointed out in our previous study, is a unit defined by topological structures [58]. However, in real-world scenes, the topological structure of the visual input is not as obvious as in simple geometric figures because local features are entangled. When these real-world scenes are inputted into deep neural networks designed for category classification, such as AlexNet, each picture can be represented by a point in a high-dimensional space, where each axis represents a given category. The closer a point is to the center of a category, the more likely it is that the picture belongs to that category. At the same time, it is more likely to be away from the centers of other categories, thus improving the possibility of its emergence as an object in contrast to its surroundings in real-world scenes. This idea was further confirmed by the inferior performance when we applied the same procedures on the last convolutional layer (see Fig. S10), indicating an integration beyond local features is necessary for numerosity perception. These results consistent with our assumption that those non-preferred responses as sidebands carried more information than the category label and the activation pattern of the classification layer for potential objects determined how likely the objects to be detected by the number system. We evaluated this possibility as an embedding coefficient and demonstrated that a higher embedding coefficient leads to more optimized performance in terms of numerosity perception.

For certain objects, the best fit category determined by the embedding coefficient differs from their category labels in the database. This can be explained by three reasons. First, the best-fit category was determined by group coding via the embedding coefficient, whereas the conventional approach used the top one or five units in the classifier layer. Second, even the original ImageNet method had an error rate of 37.5% [20], indicating misclassifications of approximately one-third of the objects. Third, there are fewer categories in the MS-COCO dataset than in ImageNet, although we believe that the underlying relationships across the categories should be similar for the two datasets if they both represent our general understanding of the real world. The more optimized performance of scenes with higher embedding coefficients confirms that despite the presence of misclassified units, it is more important to segregate them from the background for the perception of numerosity rather than correctly identifying the object. As the two datasets were designed and labeled by two groups of people separately, similar to the similarity of semantic relations found in AlexNet and WordNet, this identity-free capacity of numerosity perception may be rooted in training with the category labels that represent the inner structure of human perception across groups of people. This assumption, on the one hand, could be useful in further applications of DNN involving human perception; on the other hand, it presents an opportunity for exploring human perception through the dissection of the DNN structure and its performance.

As a striking but straightforward result, we demonstrated that for humans, real-world scenes with higher embedding coefficients showed more optimized performance in a numerosity discrimination task where the participants were unlikely to make their decisions by counting because of the short duration of the display of the pictures. Despite the fact that humans can detect numerosity in real-world scenes, which is consistent with previous studies on complexity perception [54], the more optimized performance of a superior embedded subset revealed that the high-dimensional space where those scenes are embedded, though derived from a DNN, may represent part of the infrastructure of human perception in the brain.

Compared to counting and precise object recognition, this type of representation leads to a quick and rough estimation of numerosity in real-world scenes and does not need to play a major role in ordinary visual perception. It is not obvious as an evolutionary vestige [78] with ecological functions similar to those found in hunting spiders [39,40], and may occur in the early phase of visual processing, which would contribute to guiding further detailed perception of object identities and numbers.

### 3.1 Limitations of the study

This study focuses on the capacity of DNN to detect numerosity in real-world scenes and their corresponding representations. Although the study provides results on the advantages of pictures with superior embedding shared by DNN and humans, it does not address how embedded representations emerge in the two types of agents through training or development. By enhancing the weighting of specific categories, such as faces, or by excluding certain categories from the training datasets, future studies could provide insight into the developmental mechanisms underlying this processing in the brain and artificial neural networks.

## 4 STAR methods

Key resources table.

**Table:** 

REAGENT or RESOURCE	SOURCE	IDENTIFIER
Deposited data		
Raw data and custom codes	This paper	Mendeley Data, V1, https://doi.org/10.17632/8j7fwfk8g3.1
ImageNet ILSVRC2012 dataset	Russakovsky et al., 2015	http://image-net.org/challenges/LSVRC/2012/
MS COCO dataset	Lin et al., 2014	https://arxiv.org/abs/1405.0312
Software and algorithms		
Python 3.9.13	Python Software Foundation	https://www.python.org/
Numpy 1.21.6	Open-source community project	https://numpy.org/
SciPy 1.9.1	Open-source community project	https://www.scipy.org/
Matplotlib 3.5.2	Michael Droettboom et al.	https://matplotlib.org/
PyTorch 1.12.1	Facebook's AI Research lab (FAIR)	https://pytorch.org/
scikit-learn 1.0.2	Pedregosa et al., 2018	https://scikit-learn.org
OpenCV 4.6.0	Bradski, 2000	https://opencv.org/
TensorFlow	Abadi et al., 2016	https://arxiv.org/abs/1603.04467
Keras	Chollet, 2015	https://github.com/keras-team/keras
Pycocotools 2.0.6	Lin et al., 2014	https://github.com/ppwwyyxx/cocoapi
Pands 1.4.4	NumFOCUS sponsored project	https://pandas.pydata.org/
Statsmodels 0.13.2	Open-source community project	https://www.statsmodels.org/
DNNBrain	Chen et al., 2020	https://github.com/BNUCNL/dnnbrain
PsychoPy	Open Science Tools Ltd.	https://www.psychopy.org/
RSAtoolbox	Nili et al., 2014	http://www.mrc-cbu.cam.ac.uk/methods-and-resources/toolboxes/license/

## 5 Resource availability

### 5.1 Lead contact

Further information and reasonable requests for resources and reagents should be directed to and will be fulfilled by the lead contact, Liu Zuxiang (zxliu@ibp.ac.cn) or Xu Qin (xuqin@ahu.edu.cn).

### 5.2 Materials availability

This study did not generate new unique reagents.

### 5.3 Data and code availability

● The data can be downloaded from the following repository: Mendeley Data, V1, https://doi.org/10.17632/8j7fwfk8g3.1
● The code scripts can be downloaded from the following repository: Mendeley Data, V1, https://doi.org/10.17632/8j7fwfk8g3.1

## 6 Method details

### 6.1 Neural network model

AlexNet pre-trained for object classification on the ILSVRC2012 database (version of weighting: alexnet-owt-7be5be79. pth) was used. For comparison, an untrained version of AlexNet with random weights was investigated.

### 6.2 Stimulus datasets

For experiments with mosaic pictures, the pictures used in this study were generated from the ILSVRC2012 dataset (http://image-net.org/challenges/LSVRC/2012/). The ILSVRC2012 training dataset contains nearly 1.2 million images with labels from 1000 categories, whereas the validation dataset contains 50,000 images belonging to the same 1000 categories. Only validation datasets were used in this study.

For the mosaic pictures comprising sub-blocks from the same category (numbers 2 to 7), 250 pictures were generated for each of the 1000 categories. The sub-blocks were randomly selected from 50 original pictures of a given category and placed in a grid-like layout in the composed picture (Fig. 1B). The height and width of the composed picture were determined using the maximum height and width of the sub-blocks, whereas the background was black. Therefore, there were 6 × 250 composite pictures and 50 original pictures in each category.

For the mosaic pictures comprising sub-blocks from different categories, 10,000 pictures were generated for each number from 2 to 7, and each of the sub-blocks was randomly selected from the validation datasets. For number 1, 50,000 original pictures are used. To test whether the layout of the sub-blocks on a black background determined the patterns of the group activations, a noise patch version of these pictures was created by replacing the contents of the sub-blocks with white noise.

For pictures of real-world items (Fig. 2A), the size of the pictures is 2000 × 2000 pixels, with a black background. Objects from the ILSVRC2012 validation dataset were first clipped from the original picture according to their rectangular bounding box and masked by an ellipse inscribed to the bounding box. The elliptical patch was rescaled to an appropriate size, as determined by the algorithm described below. Before being pasted onto the picture, an inverted Gaussian filter with a size of 15 × 15 pixels was applied along the edge of the ellipse to obtain a soft gradient on the black background. The total area occupied by the objects in the picture was set to approximately 250 × 250 × 7 pixels, and the average size of the objects was obtained by dividing the total area by the number of items in the picture. However, we applied random scale factors within the range of ±25% to the size of the objects to balance the tradeoff between the total area and size of the objects. For each picture with more than 2 items, 1000 combinations of these scale factors were evaluated and the optimal combination that matched the preset total area was used. The positions of the objects are determined by dividing the picture into 4 × 4 virtual grids (grid size = 500 pixels) and randomly assigning objects to the grids. The position of the object was then fine-tuned by random jittering within the grid to ensure that it did not overlap with other objects in the picture. This design compensated for the trade-off between the density and number of objects. Three datasets were generated using the same procedure: (a) objects in the picture are randomly from different categories, (b) objects in the picture are from the same category, and (c) the ellipses in the different category conditions were filled with white noise before the inverted Gaussian filter was applied to the edge.

The MS COCO datasets were used for experiments involving real-world scenes (https://cocodataset.org/#home). The MS COCO datasets contain approximately 328 K images, where common objects in context are labeled using bounding boxes with a category tag. Both the training and validation datasets were used. From the MS COCO training dataset, pictures with one bounding box were categorized as number 1 and pictures with 2 bounding boxes as number 2, in that order until pictures with 7 bounding boxes were categorized as number 7 (Fig. 3A). The training set (COCO_train) was generated by randomly choosing 4000 pictures from each of the seven groups. Another 7 groups of pictures for validation (COCO_val) were generated in the same manner, with each having 150 pictures from the MS COCO testing dataset.

Several modified versions of the pictures in the COCO_val were generated: (1) COCO_val with noise, where 50%, 100%, 200%, and 300% weighting of white noise were added to the respective original pictures; (2) COCO_val_clip, where the area outside bounding ellipses followed the same procedure as described above, was shaded black; (3) noise clip, in which the area outside of bounding ellipses was shaded black and the area within the bounding ellipses was filled with white noise; (4) COCO_val_bbox_clear, where the area inside of bounding ellipses was shaded black; (5) COCO_val_scrambled, where the pictures were first converted to frequency domain via Fourier transform, followed by shuffling of the phase section before they were converted back to spatial domain by inverse Fourier transform. For all the pictures where the bounding ellipses were involved, the inverted Gaussian filter, as described above, was applied to obtain a soft edge.

### 6.3 Analysis of the responses of the network units

Responses to each picture were extracted from all 1000 units in the last fully connected layer using the DNNBrain toolbox [79]. The responses were then normalized from 0 to 1 using the minimum and maximum responses among the 1000 units.

To evaluate the changes in activation with the number of items, 500 pictures were randomly selected from the datasets (mosaic pictures, pictures comprising real-world items, and MS-COCO). The mean value and standard deviation of the responses across 500 pictures were calculated for each unit. The 9th decile (90%) of the mean activations (Fig. 1E, left) or the 1st decile (10%) of the standard deviations across the 1000 classifier units were measured. The same procedure was repeated 10 times for every dataset, and statistical tests were applied based on the distribution of the activations or the 9th or 1st decile of the activations.

### 6.4 Decoding numerosity from group activations

Based on the activations to the pictures in the COCO_train dataset, decreasing and increasing units were selected from the 1000 output units with the following criteria: increasing or decreasing activations along with the number of objects in the pictures; the difference in activations has a p value below 0.1 for every consecutive number pair. There were 48 increasing units (1 item < 2 items < … < 7 items) and 13 decreasing units (1 item > 2 items > … > 7 items).

A simple decoding network was designed by considering the activations in the aforementioned 61 units as inputs, followed by a fully connected hidden layer of 2000 units and a fully connected output unit. The response of the output unit is assumed to be the estimated number of objects in the picture. The decoding network was trained using the COCO_train dataset (4000 × 7 pictures) and evaluated using both the COCO_train and COCO_val datasets (150 × 7 pictures). The trained decoding network was also applied to tests involving COCO_val and other modified versions of the dataset.

### 6.5 Numerosity comparison task for the network

A numerosity comparison task was adopted from previous studies [27,31] to examine the performance of the decoding network in estimating numerosity from pictures. For each trial, a sample and test picture were presented to the network, and the resulting responses of the output unit were recorded. A full combination of numbers from 1 to 7 was used in the design; therefore, the sample and test pictures were individually selected from 1 to 7, resulting in a 7 × 7 performance matrix. For each trial in each cell of the performance matrix, responses to the sample picture (stimulus A) and test picture (stimulus B) were used to predict whether the numerosity of stimulus A is greater than that of stimulus B. A psychometric function was calculated from the performance matrix, with numerical distances ranging from −6 to 6, by combining all the trials in proper cells (e.g., the cells in diagonal have a numerical distance of 0) in the performance matrix to obtain an averaged performance indexed as the percentage of trials that were reported as stimulus A > stimulus B.

For most tests, there were 100 trials for each cell of the performance matrix, whereas pictures were randomly selected from the 150 pictures in each of the 7 groups (from the 4000 pictures in the case of COCO_train). For the tests concerning superior embedding, there were 33 trials for each cell of the performance matrix; therefore, pictures were randomly selected from the 50 pictures with the highest embedding coefficient. For the inferior embedding condition, 33 pictures were randomly selected from the 50 pictures with the lowest embedding coefficients. The entire procedure was repeated 10 times.

### 6.6 Embedding coefficient of the pictures

For each picture in the COCO_val dataset, the contents in each bounding box were cropped and served as inputs for AlexNet. The activations in the output units were compared with those of 1000 categories to determine the optimal match. Correlation coefficients were calculated between the bounding boxes and all categories. The category with the maximum correlation coefficient was selected as the embedded category, and the coefficient was assigned to the bounding box. For a given picture, the embedding coefficient was determined by averaging the coefficients across all bounding boxes within it.

A category-based embedding coefficient was defined as follows: the bounding boxes (4200 in total for 150 × 7 pictures) were sorted into 80 categories according to their MS COCO label. For each bounding box in a given picture, the embedding coefficient is replaced with the mean coefficient of the category to which it belongs. The category-based embedding coefficient of a picture was determined by averaging the category-based coefficients across all the inherent bounding boxes.

### 6.7 Human behavioral experiment

Seventeen healthy college students (seven females; age 19–30 years, mean = 23.8, SD = 3.1; data from 5 participants were excluded owing to strong bias to the left hemifield) participated in the experiment. All the patients had normal or corrected-to-normal vision. This study was approved by the Ethics Committee of the Institute of Biophysics, CAS, and all participants provided written informed consent prior to the experiment.

A numerosity comparison task was adopted from a previous study [58]. The paradigm can be briefly described as follows: two pictures were presented in the left and right hemifields of a participant for a duration of 600 ms. The participants were instructed to identify the picture with more objects by pressing a button while maintaining consistency in their definition of/feeling about the object throughout the experiment. The experiment was conducted using PsychoPy [80].

The same design as the full combination of numbers 1–7 was applied, with 50 pictures with superior embedding and 50 with inferior embedding. A total of 7 × 7 × 50 × 2 = 4900 trials were presented in a random sequence. The participants were allowed to break when necessary. For superior and inferior embedding pictures, the 7 × 7 performance matrix and its corresponding psychometric function were calculated separately.

### 6.8 Representational similarity analysis

To compare the results from the humans with those from AlexNet, a well-established software package, RSAtoolbox [69], was applied. At the condition level, the two S-curves from the superior and inferior embedding pictures were filled into a representational similarity matrix (RSM). The RSMs from the 12 participants were used as reference RSMs, whereas the RSMs from AlexNet, the condition-shuffled AlexNet and untrained AlexNet (one instance of RSM each) were used as candidate RSM. The RSA toolbox tests the correlation between the reference RSMs and each candidate RSM and compares the difference between the averaged reference RSM and candidate RSMs. At the picture-wise level, the tests were separately applied to numbers 1–7. Within each number, the response profiles of the 100 pictures (50 in the superior embedding condition and 50 in the inferior embedding condition) were calculated by combining the results from all human participants, owing to the limited number of trials in the human behavioral experiment. A single 100 × 100 RSM of the results obtained from the participants was calculated from this response profile. As the RSA toolbox requires the reference RSMs to be a set of at least 12 RSMs, we must maintain the RSM from the participants as a candidate RSM. A similar procedure is applied to the AlexNet results. We repeated the comparison task on AlexNet 12 times to mimic 12 participants. A set of 12 RSMs from AlexNet was used as the reference RSMs. Other candidate RSMs were measured based on the results shuffled between pictures, the results from shuffled AlexNet and untrained AlexNet.

### 6.9 Responses of the last convolutional layer

To compare with the results from the classification layer, the same procedures as described above had been applied to the last convolutional layer, conv5, as a baseline condition. This layer has a structure of 256 × 13 × 13 units. Responses to each picture were extracted from all these units using the DNNBrain toolbox [79] and reshaped into a 43,264 × 1 vector before normalization. There were 16 increasing units (1 item < 2 items < … < 7 items) and 24 decreasing units (1 item > 2 items > … > 7 items) found. Normalized activations of these 40 units were fed to the decoding network, followed by a fully connected hidden layer of 2000 units and a fully connected output unit. Responses of the output unit were compared in the numerosity comparison task between pictures and conditions.

### 6.10 Statistical analysis

A one-way analysis of variance (ANOVA) was used to compare the mean and standard deviation across categories or pictures for numbers 1–7. Paired t-tests were used to select increasing or decreasing units. A two-way ANOVA was used to compare the estimated numerosity for the COCO_train and COCO_val datasets. For all numerical distances (−6–6) of the psychometric function, the one-sample *t*-test was used to compare the performance at the 50% chance level, and the paired *t*-test was used to compare any two conditions. The Bonferroni correction was applied for multiple comparisons. For the representational similarity analysis, the test between the reference RSMs and each candidate RSM was performed using a one-sided Wilcoxon signed-rank test across participants. The false discovery rate is controlled for these tests across the reference and candidate RSMs.

## Funding

Ministry of Science and Technology (China); Grant Number: 2020AAA0105601.

10.13039/501100002855Ministry of Science and Technology (China); Grant Number: 2022ZD0211901.

10.13039/501100002855Ministry of Science and Technology (China); Grant Number: 2019YFA0707103.

10.13039/501100001809National Nature Science Foundation (China); Grant Number: 31730039.

10.13039/501100001809National Nature Science Foundation (China); Grant Number: 72071001.

10.13039/501100001809National Nature Science Foundation (China); Grant Number: U21A20388.

10.13039/501100002367Chinese Academy of Sciences; Grant Number: ZDBS-LY-SM028.

Natural Science Foundation for the Higher Education Institutions of Anhui Province; Grant Number: KJ2021A0038.

## Author contribution statement

Wu Wencheng: Performed the experiments; Analyzed and interpreted the data; Contributed reagents, materials, analysis tools or data; Wrote the paper.

Yingxi Ge: Performed the experiments; Analyzed and interpreted the data; Wrote the paper.

Zhentao Zuo: Analyzed and interpreted the data; Contributed reagents, materials, analysis tools or data.

Lin Chen: Conceived and designed the experiments; Wrote the paper.

Xu Qin: Liu Zuxiang: Conceived and designed the experiments; Analyzed and interpreted the data; Wrote the paper.

## Data availability statement

Data associated with this study has been deposited at Mendeley Data, V1, https://doi.org/10.17632/8j7fwfk8g3.1, https://data.mendeley.com/datasets/8j7fwfk8g3/1.

## Declaration of competing interest

The authors declare that they have no known competing financial interests or personal relationships that could have appeared to influence the work reported in this paper

## Abbreviations

DNNs: deep neural networks
RSMs: representational similarity matrices
WordNet: a large lexical database of English
AlexNet: a classic convolutional neural network architecture
LSVRC2012: The Large Scale Visual Recognition Challenge 2012 dataset
MS COCO: Microsoft Common Objects in Context dataset
COCO_train: training dataset from MS COCO
COCO_val: test dataset from MS COCO
COCO_val_clip: test dataset, background outside of focus regions is filled with black
noise_clip: test dataset based on COCO_val_clip, focus regions are filled with white noise
COCO_val_bbox_cleared: test dataset, focus regions are filled with black
COCO_val_scrambled: test dataset, scrambled in phase domain
ANOVA: one-way analysis of variance
