# Supplementary material for: Visual number sense for real-world scenes shared by deep neural networks and humans
Source: Heliyon. 2023 Jul 24;9(8):e18517. doi: 10.1016/j.heliyon.2023.e18517 (PMC10407052; doi:10.1016/j.heliyon.2023.e18517)
Supplement: Multimedia component 1 [file mmc1.pdf]

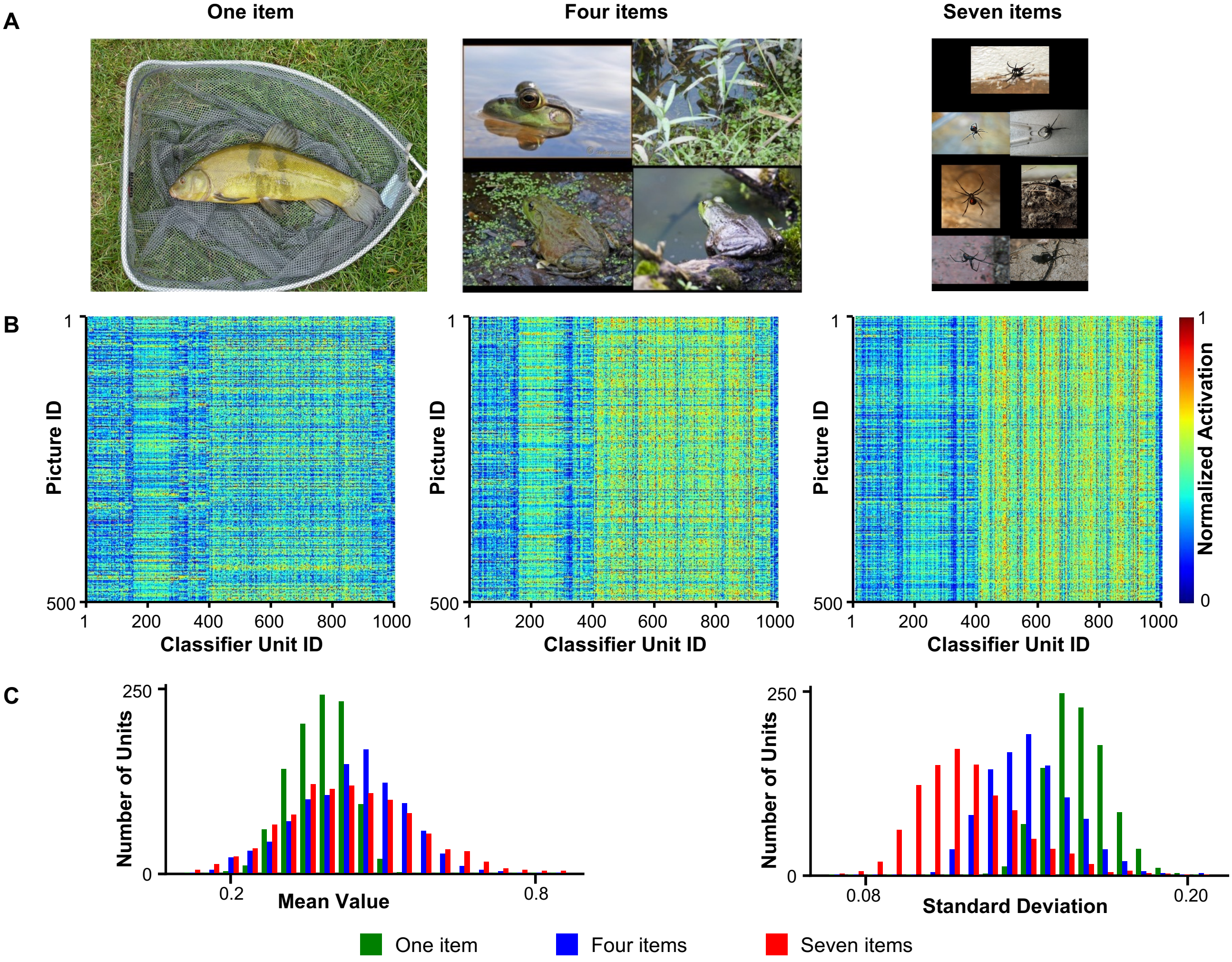

Figure S1. Group activations change with the number of items from the same category.
(**A**) Sample mosaic pictures. Left: a single picture from ImageNet; middle: a mosaic picture composed of four items from the same category; right: a mosaic picture constituting seven items. (**B**) Activation maps for mosaic pictures that have different numbers of items. From left to right, the intensity generally increases from item 1–7, and the co-activation pattern is shared as vertical lines across the pictures. (**C**) Left: with more items in the picture, more units pitch at higher activations; right, significant shared co-activations increase with the number of items, characterized as more units have smaller standard deviation of activation.

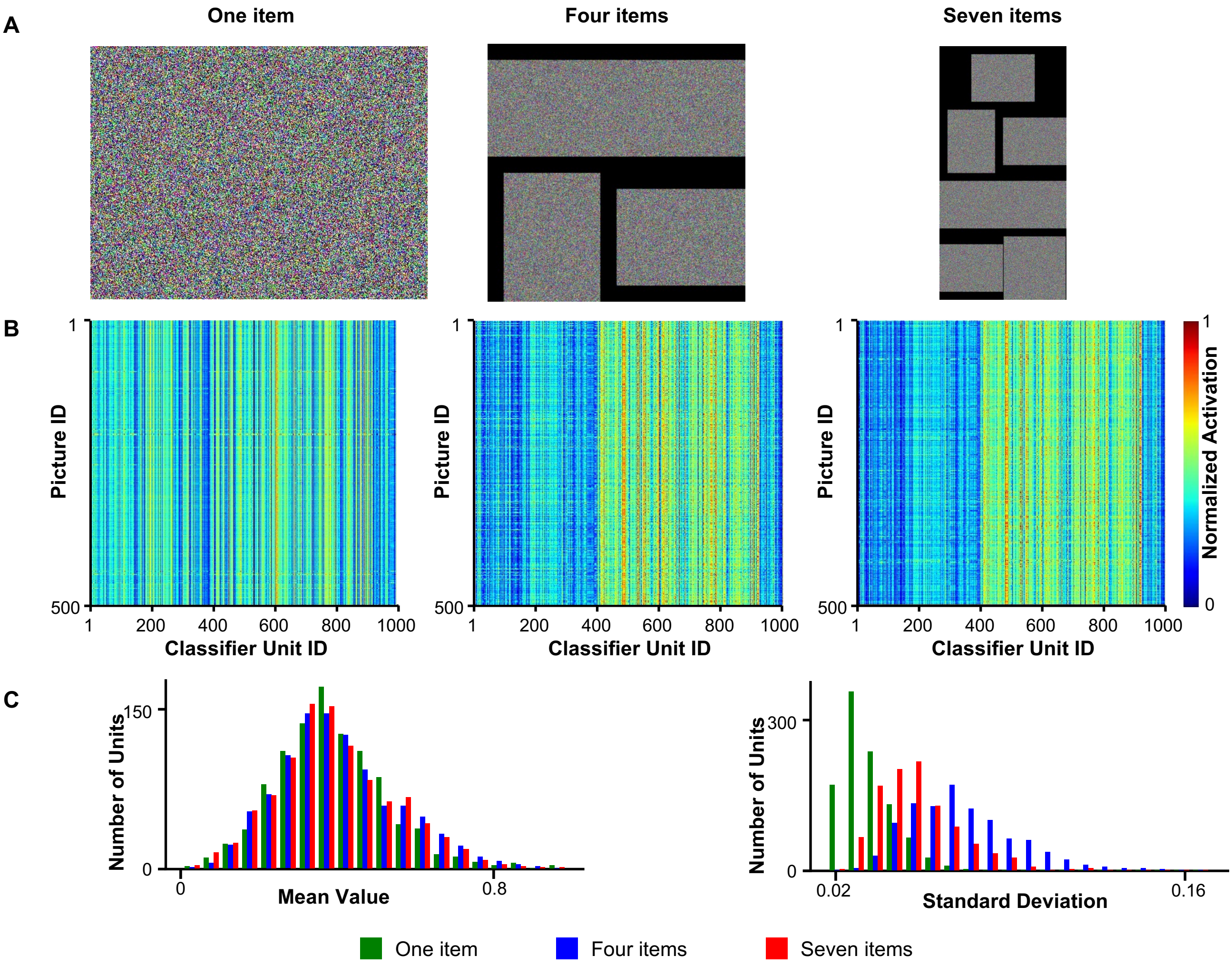

Figure S2. Group activations change with the number of noise patches.
(**A**) Sample mosaic pictures filled with white noise. (**B**) Activation maps for mosaic pictures that have different numbers of items. The intensity and co-activation pattern changed with the number of items. (**C**) Left: with more items in the picture, more units pitch at higher activations; right: pictures with a single item have the smallest standard deviation of activation.

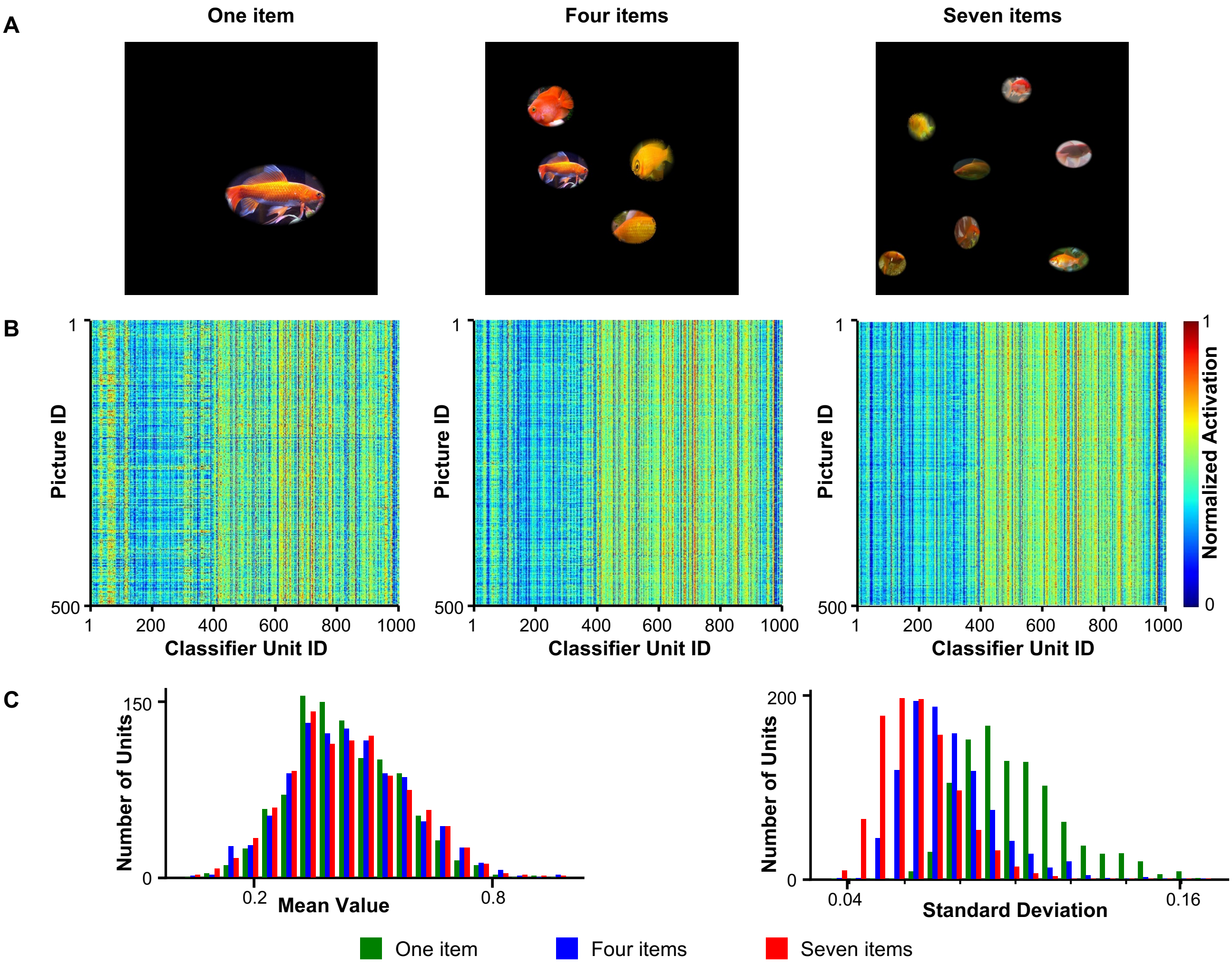

Figure S3. Numerosity represented by group activations for real-world items from the same category.
(**A**) Sample visual stimuli with real-world items. Left: a single item; middle: four items from the same category; right: seven items from the same category. (**B**) Activation maps for pictures with different numbers of items. From left to right, the intensity increased from one item to seven items and the shared co-activation pattern as vertical lines across the pictures was obvious. (**C**) Left: with more items in the picture, more units pitch at higher activations; right: significant shared co-activations increase with the number of items, characterized as more units having a smaller standard deviation of activation.

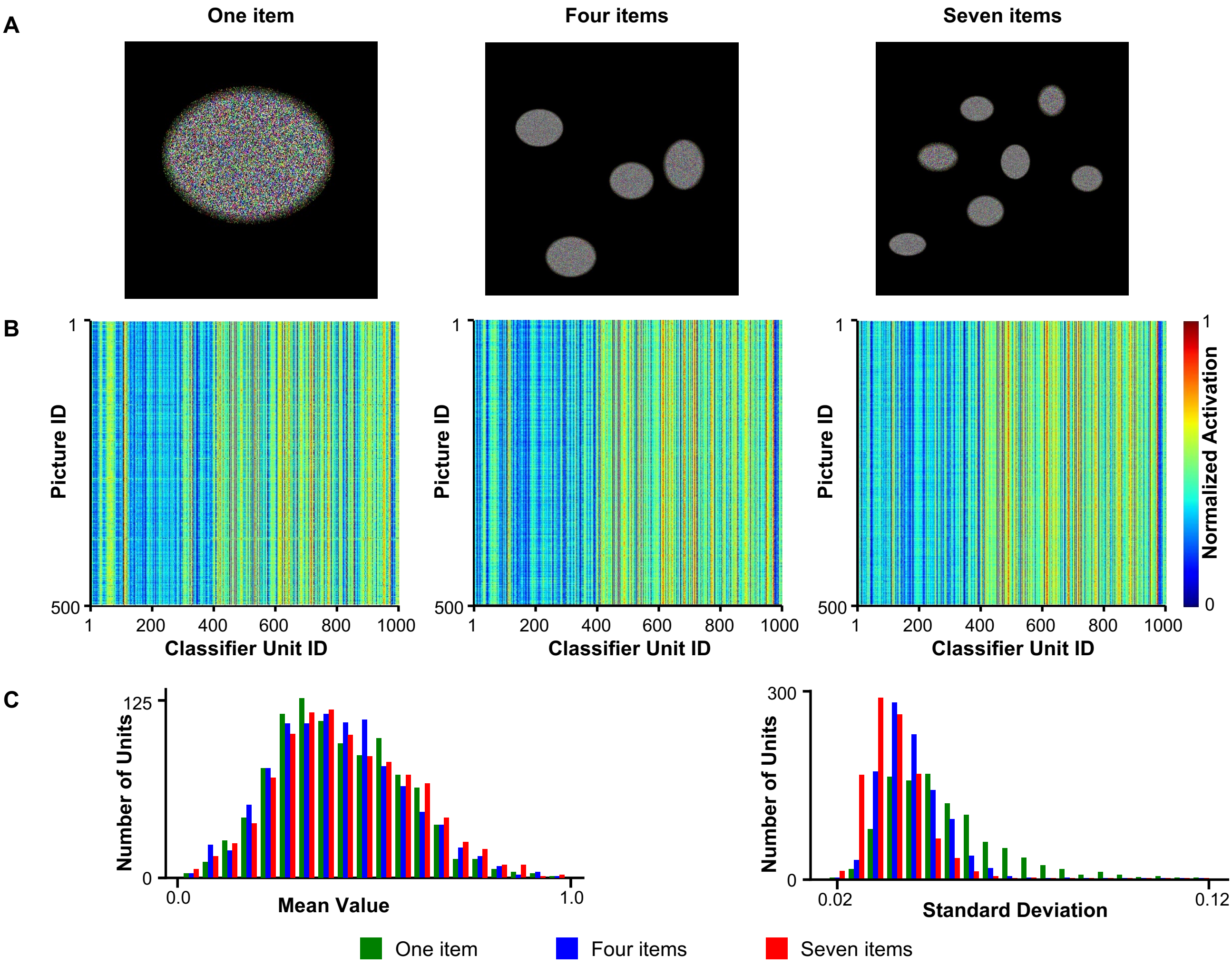

Figure S4. Numerosity represented by group activations for elliptical noise patches.
(**A**) Sample visual stimuli with elliptical noise patches. (**B**) Activation maps for pictures with different numbers of items. From left to right: the intensity increased from one to seven items and the shared co-activation pattern as vertical lines across the pictures was obvious. (**C**) Left: with more items in the picture, more units pitch at higher activations; right: significant shared co-activations increase with the number of items, characterized as more units having a smaller standard deviation of activation.

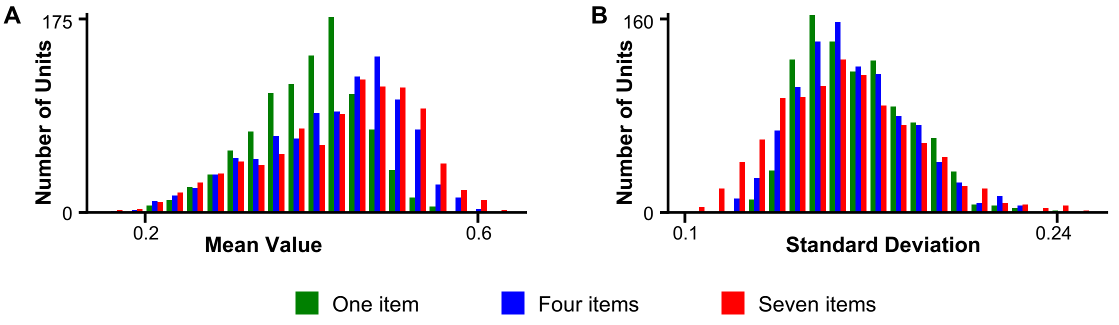

Figure S5. Same activation pattern found in the COCO testing dataset.
(**A**) Histogram of mean activations for the 1000 classifier units. Larger activation for pictures with 7 items. (**B**) Histogram of standard deviations. Smaller standard deviation for pictures with 7 items.

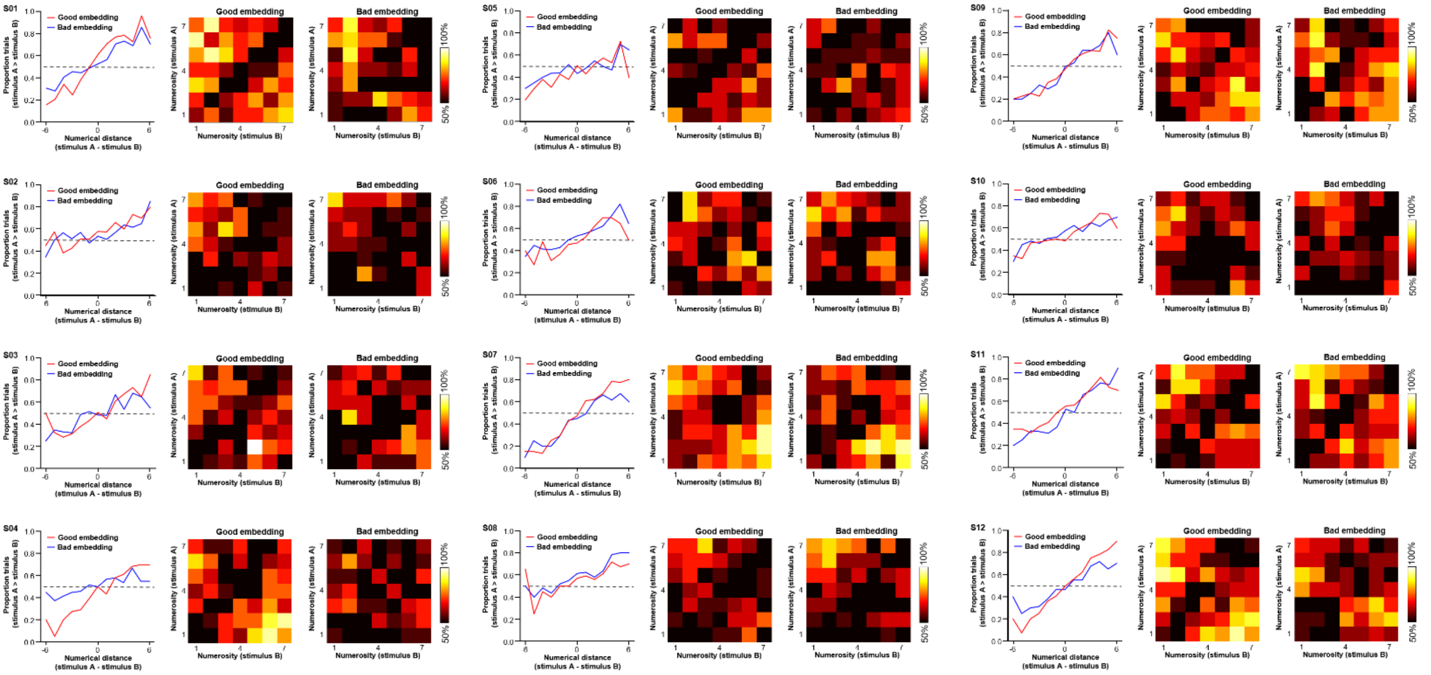

Figure S6. Psychometric function and performance matrix for individual participants (N=12).

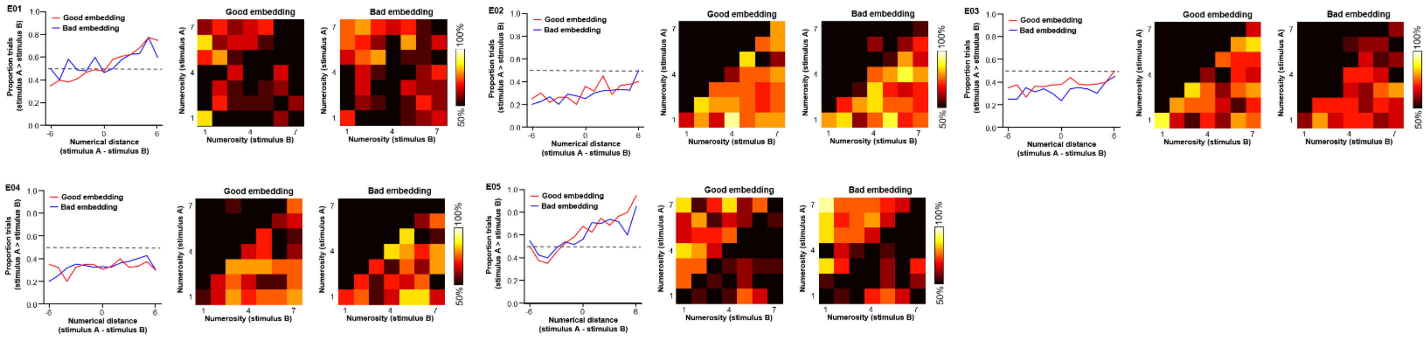

Figure S7. Psychometric function and performance matrix for the five participants excluded from the study.

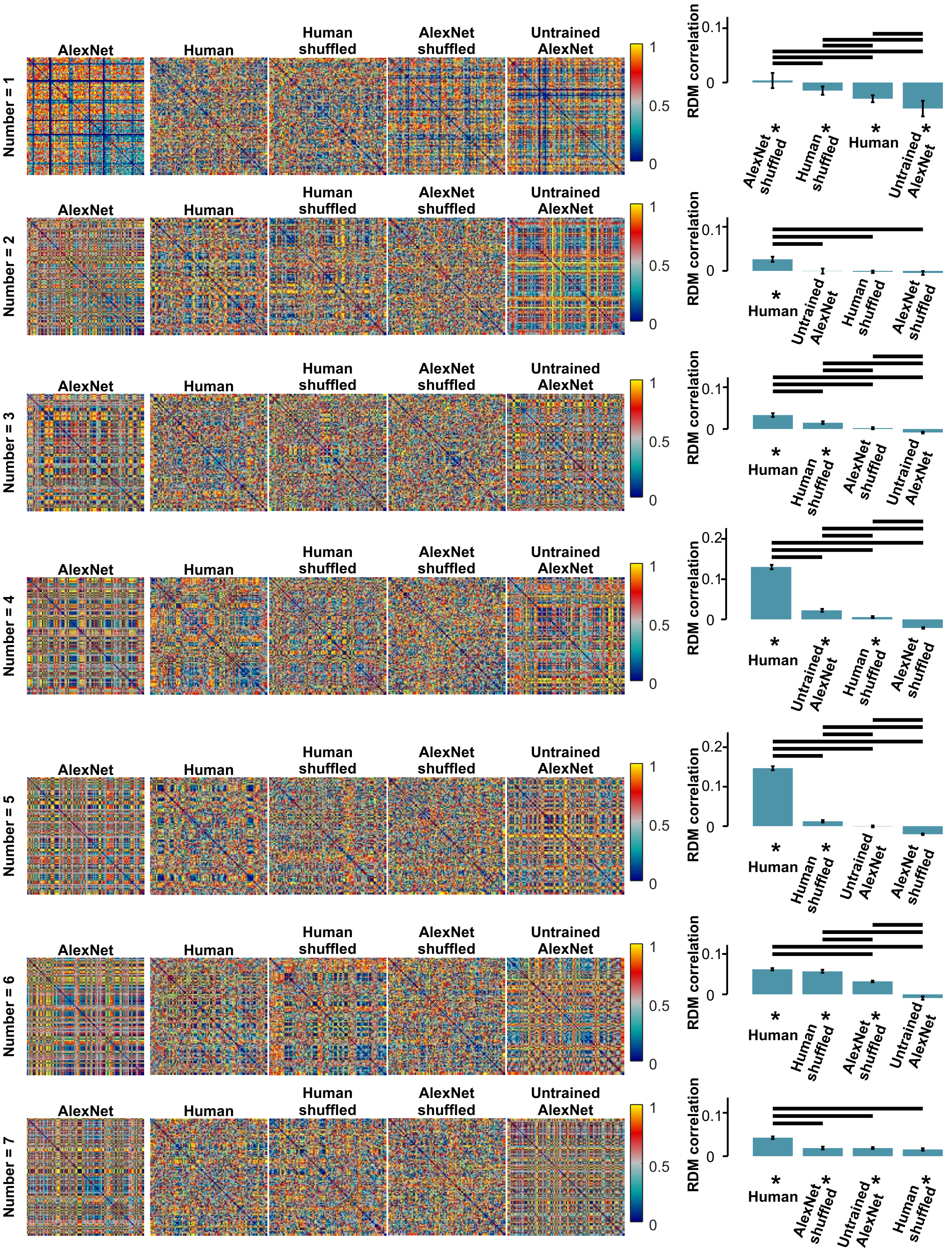

Figure S8. Representational similarity analysis at picture level. Left: RDMs for AlexNet, Human behavioral responses and other control conditions. Right: AlexNet was compared with other conditions and there were significant similarities to human performances from numbers 2 to 7.

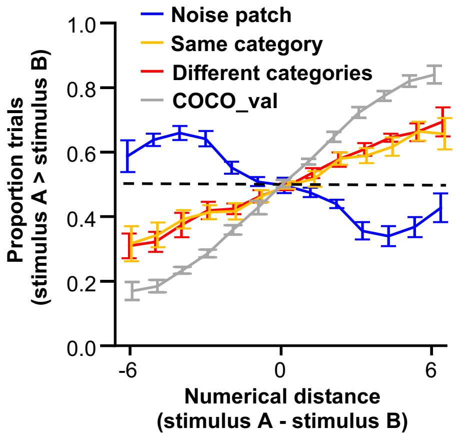

Figure S9. Performance of comparison task for pictures composed of real-world items. Regardless of the real-world items being from the same or different categories, similar results were obtained, indicating that neither the confidence of the existence of a given category nor the diversity of categories played a major role in representing the number of objects.

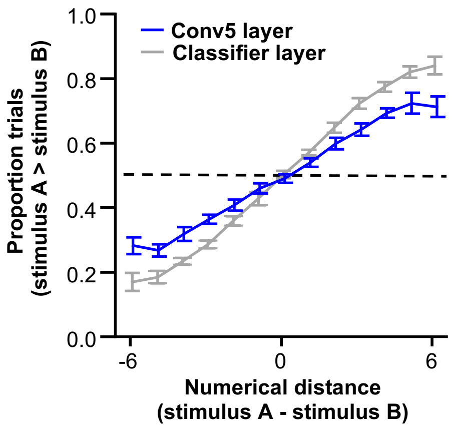

Figure S10. Performance of comparison task for the classifier layer and the final convolutional layer. When the same procedure was applied to the final convolutional layer, the layer with most number-selective neurons (conv5), the results were significantly worse than those obtained from the classifier layer, indicating that the number sense for real-world scenes represents some innate information beyond the local features of visual inputs.
